# Improved Ferroelectric Properties in Hf_0.5_Zr_0.5_O_2_ Thin Films by Microwave Annealing

**DOI:** 10.3390/nano12173001

**Published:** 2022-08-30

**Authors:** Biyao Zhao, Yunting Yan, Jinshun Bi, Gaobo Xu, Yannan Xu, Xueqin Yang, Linjie Fan, Mengxin Liu

**Affiliations:** 1 Institute of Microelectronics, Chinese Academy of Sciences, Beijing 100029, China; 2 College of Integrated Circuits, University of Chinese Academy of Sciences, Beijing 100049, China; 3 Boston University, Boston, MA 02215, USA; 4 Beijing Zhongke New Micro Technology Development Co., Ltd., Beijing 100029, China

**Keywords:** ferroelectricity, remanent polarization (Pr), TiN/Hf_0.5_Zr_0.5_O_2_/TiN film, wake-up effect, leakage current

## Abstract

In the doped hafnia(HfO_2_)-based films, crystallization annealing is indispensable in forming ferroelectric phases. In this paper, we investigate the annealing effects of TiN/Hf_0.5_Zr_0.5_O_2_/TiN metal-ferroelectric-metal (MFM) capacitors by comparing microwave annealing (MWA) and rapid thermal annealing (RTA) at the same wafer temperature of 500 °C. The twofold remanent polarization (2Pr) of the MWA device is 63 µC/cm^2^, surpassing that of the RTA device (40 µC/cm^2^). Furthermore, the wake-up effect is substantially inhibited in the MWA device. The orthorhombic crystalline phase is observed in the annealed HZO films in the MWA and RTA devices, with a reduced TiN and HZO interdiffusion in MWA devices. Moreover, the MFM capacitors subjected to MWA treatment exhibit a lower leakage current, indicating a decreased defect density. This investigation shows the potential of MWA for application in ferroelectric technology due to the improvement in remanent polarization, wake-up effect, and leakage current.

## 1. Introduction

Ferroelectric (FE) materials based on doped-hafnia(HfO_2_), particularly Hf_0.5_Zr_0.5_O_2_ (HZO) thin films, emerge as promising candidates for extensive applications in the non-volatile memory, logic, and neuromorphic devices owing to their superior properties of good scalability and full CMOS-compatibility [1,2,3,4,5]. The crystallographic phases in HZO films include the tetragonal (P4_2_/nmc, t-phase), orthorhombic (Pca2_1_, o-phase), and monoclinic phases (P2_1_/c, m-phase) [6,7,8,9,10], wherein the non-centrosymmetric o-phase is considered to be the origin of ferroelectricity. However, the as-deposited HZO films are generally weak, even non-ferroelectric, and the annealing process is indispensable for crystallizing the orthorhombic phase.

Rapid thermal annealing (RTA) is the most commonly used process in the manufacture of ferroelectric devices [11,12]. However, the RTA process can also degrade the electrode/FE interface and increase the interface state density (*D_it_*), leading to an undesirable built-in electric field and the wake-up effect during the subsequent electric cycles [13,14,15,16,17,18]. This is detrimental for ferroelectric-based technologies applied in logic devices requiring stable and superior remanent polarization (Pr) values. On the other hand, microwave annealing (MWA) [19,20,21,22,23] is considered an alternative annealing technique with the advantage of mitigating the annealing-induced defects.

The MWA treatment has been studied in several other material systems, including HfO_2_-based MOS capacitors [24], Ni(Si,Ge) films [25], and PZT films [26]. During the annealing process, the HZO films are exposed to the microwave environment that can provide energy for the molecules’ rotation and polarization. The state of the polarization rotation would change with the loading of a cyclical electric field, that is, the wake-up effect, which decisively determines the performance of ferroelectric-based memory devices. Compared with the RTA process, the MWA process has superior properties with respect to the lower thermal budget [27], better interface state, and less dopant diffusion. Furthermore, it is promising to alleviate the wake-up effect and leakage current, and there are no relevant studies.

In this paper, we investigated the promoting effect of the MWA process on TiN/HZO/TiN metal-ferroelectric-metal (MFM) capacitors through the combination of experimental data and physical mechanisms. Moreover, to validate our perspective, identical capacitors with identical structures and front-end processes annealed by RTA were performed as the control group.

## 2. Device Design and Fabrication

The TiN/HZO/TiN MFM capacitors were manufactured on 8-inch heavily-doped p-type silicon wafers. The natural surface oxidation was removed by etching with a diluted HF solution. The top and bottom 20 nm TiN electrodes were deposited by radiofrequency (RF) reactive magnetron sputtering at room temperature. A metallic Ti target and a mixture of Ar and N_2_ gas were utilized in the deposition, with the sputtering power of 2 kW and N_2_ gas flow rate of 8 SCCM. The 10 nm of HZO films were deposited in situ by thermal atomic layer deposition (ALD) at a substrate temperature of 280 °C. During the deposition, the precipitation source of Hf, Zr, and oxygen were Hf[N(C_2_H_5_)CH_3_]_4_, Zr[N(C_2_H_5_)CH_3_]_4_, and H_2_O, respectively. At this point, the front-end process of the MFM capacitors had been completed. Afterward, the as-deposited samples were divided into two groups to conduct the MWA and RTA treatments. The MWA samples were annealed in a DSGI octagonal MWA chamber in N_2_ ambient with an excitation frequency of 5.8 GHz and a magnetron power of 3600 W for 30 s. The annealing temperature was maintained at about 500 °C, monitored by a line-of-sight infrared pyrometer on the wafer backside. For comparison, the other group was annealed with an ordinary RTA process performed at 500 °C for 30 s in N_2_ ambient.

Firstly, we measured the polarization-voltage (P-V) and current-voltage (I-V) characteristics of the as-deposited (without post-metallic annealing), MWA, and RTA devices by the Radiant Workstation ferroelectric tester (Radiant Technologies, Inc., Albuquerque, NW, USA) and Agilent B1500 semiconductor parameter analyzer (Agilent, Santa Clara, CA, USA). Furthermore, the grazing-incidence X-ray diffraction (GIXRD) measurement was conducted (1W1A Diffuse X-ray Scattering Station of Beijing Synchrotron Radiation Facility, Beijing, China), with Cu Kα radiation and an incident angle of 1° to examine the crystal structures of the HZO thin films. In addition, the cross-sectional structures and components were analyzed by standard and high-resolution transmission electron microscopy (HRTEM) (Thermo Fisher Scientific, Waltham, MA, USA) and Energy Dispersive X-ray (EDX) spectroscopy (Thermo Fisher Scientific, Waltham, MA, USA).

## 3. Results and Discussion

**Figure 1:**
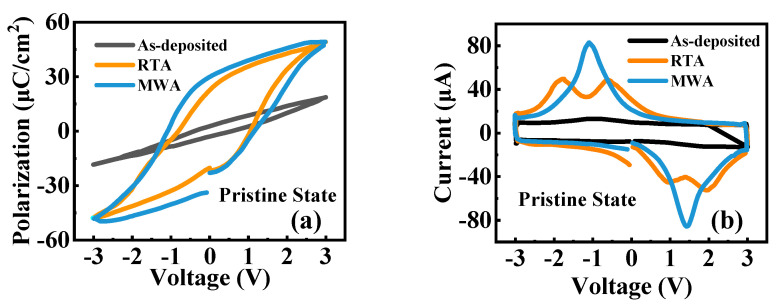
Ferroelectric characteristics of the as-deposited, RTA, and MWA devices. (**a**) P-V hysteresis loop plot. (**b**) I-V characteristics.

Figure 1a,b shows the P-V hysteresis loop and the I-V characteristics under different annealing treatments of the MWA, RTA, and as-deposited MFM capacitors in their pristine states. Accordingly, the as-deposited capacitor exhibits weak ferroelectric properties. In Figure 1a, the capacitor subjected to RTA treatment shows ferroelectric hysteresis with a 2Pr value of 40 µC/cm^2^ and a coercive field of 1 MV/cm, consistent with the data reported in the previous studies [12,15,28,29]. The MWA capacitor exhibits the strongest ferroelectric properties for the corresponding 2Pr value of 63 µC/cm^2^, exceeding that of the RTA counterpart. The RTA capacitor in Figure 1b is in a non-woken-up state. The I-V characteristics exhibit two separate peaks on each side of the applied voltage, primarily attributed to the defect-related domain wall pinning and seed inhibition [13,16]. The local defects, such as oxygen vacancy (*V*_O_) in the ferroelectric film, led to a higher Gibbs-energy barrier and difficulties in polarization switching [16]. In contrast, in the MWA curve, a single peak with a higher intensity is observed on each side, indicating the suppression of the domain wall pinning and seed inhibition in the MWA capacitor.

**Figure 2:**
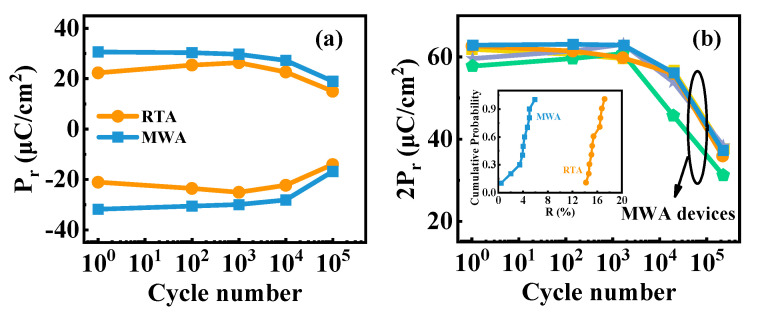
Ferroelectric characteristics of the RTA and MWA devices. (**a**) Electric field cycles performance. (**b**) Twofold remanent polarization (2Pr) versus the electric field cycles of the MWA capacitors. The inset is the statistical plot of the degree of wake-up (*R*) effect.

To completely understand the effects on the endurance of MFM capacitors subjecting to different annealing treatments, the remanent polarization (Pr) performance with electric field cycles was measured, as illustrated in Figure 2a. For the MFM capacitors that underwent RTA, Pr shows two distinct successive trends with increasing cycles, known as the “wake-up” and “fatigue” effect [16,17,18]. When the cycles are less than 10^3^, Pr increases with the cycles until it reaches the maximum, known as the “wake-up” effect. Subsequently, the RTA capacitor gets into the “fatigue” stage as the Pr value decreases with further electric field cycling. Unfortunately, the undesirable endurance characteristics with unstable Pr value cannot satisfy the high-precision requirements in logic and memory applications. The MWA capacitor also shows a fatigue effect after 10^3^ cycles. However, in contrast with the RTA capacitor, the Pr values of the MWA capacitor remain virtually unchanged during electric field cycles before the “fatigue” stage occurs, exhibiting excellent “wake-up-free” properties. 

Figure 2b shows the cycling results of several other samples subjected to the MWA treatment, which exhibit outstanding 2Pr values of ~60 µC/cm^2^ in the pristine state. To quantify the extent of the change in the 2Pr value, we introduced the parameter *R*, defined as:

(1) $$R=\frac{\left|P_{1000}-P_{1}\right|}{P_{1}}\times 100\%$$

where *P*_1_ and *P*_1000_ are the 2Pr values at the cycle numbers 1 and 1000, the inset of Figure 2b shows the cumulative statistical possibility of *R* values. As illustrated, the *R* distribution falls at around 15% of the RTA capacitors. In contrast, those of the MWA counterparts are below 5%, indicating that the MWA process can effectively weaken the wake-up effect.

MWA differs from RTA during the annealing process primarily due to the rapidly changing electric fields [18] that act on the HZO film, which has been demonstrated as an influential factor in inducing a stable ferroelectric phase [2]. When the electric field is oriented in the o-phase polarization direction, the free energy of the polar phase will decrease more than that of the nonpolar phase. The first-principle calculations [30] of the density functional theory (DFT) have suggested that the optimum endurance of the ferroelectric phase in hafnia films can be obtained with the comprehensive electric field and other relevant factors. In the samples investigated in this work, there are several optimized factors facilitating the superior ferroelectric properties, such as the capping of the TiN top and bottom electrodes, the 50% Zr doping concentration, and the 10-nm thickness of the HZO film [16,31] (detailed information are provided in Figure S1 of Supplementary Information).

**Figure 3:**
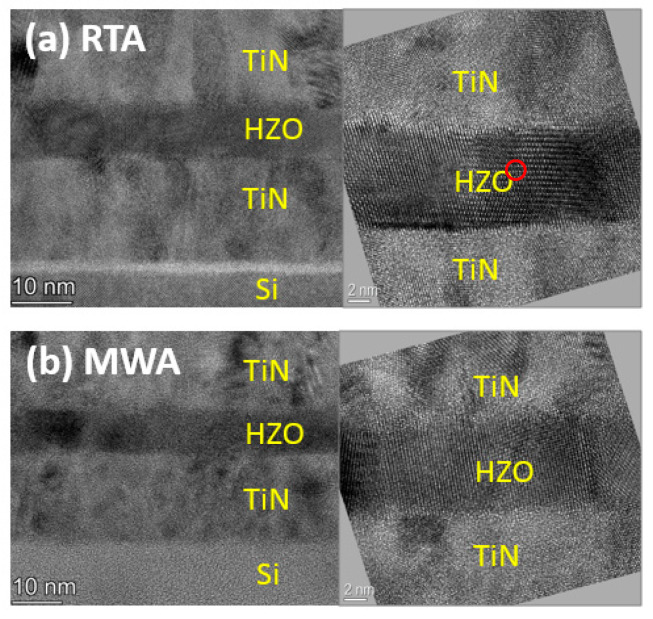
The cross-sectional TEM images with measured lattice distances of the (**a**) RTA and (**b**) MWA samples. The circled region in the RTA sample denotes discoloration.

The ferroelectric phases can be characterized quantitatively through grazing-incidence X-ray diffraction (GIXRD) measurement, which is also reflected in the lattice fringes. Through physical properties and lattice structural analysis, it can be identified that the HZO crystallites consist of a mixture of o- and m-phases. As the HRTEM images show in Figure 3, the gradual deviation from bulk values of HZO lattice fringes can be observed in both RTA and MWA samples, reflecting the presence of stress field and strain [32]. In the RTA sample, obvious dislocation is generated to accommodate the stress field, accompanied by a continuous strain deformation. It is considered that the RTA sample is with more deformations and bigger strain compared to the MWA counterpart.

**Figure 4:**
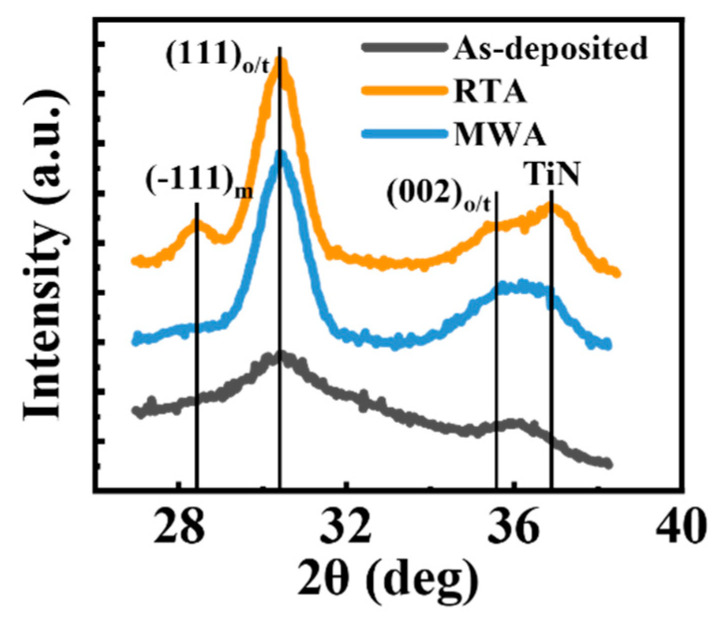
The GIXRD spectra of the annealed HZO films treated with MWA and RTA treatment and the as-deposited sample.

The GIXRD measurement was performed on the 10-nm annealed HZO films subjected to RTA, MWA, and the as-deposited status to analyze the crystal structure. As shown in Figure 4, a set of diffraction peaks attributed to the HZO film with 2θ at 28.5°, 30.5°, and 35.5° are characterized. The peak at 28.5°, corresponding to the most intensive (−111) m-phase reflections, can be obviously seen in the spectrum of the RTA device, but it is not as evident in the spectrum of the MWA device. Although the disappearance of the monoclinic (−111) diffraction peak cannot absolutely validate the absence of the m-phase in the MWA device, the experimental results can still illustrate that the MWA process exhibits a higher efficiency than the RTA process due to the induced electric field in annealing. The MWA sample demonstrates better ferroelectric properties with higher Pr values than the RTA counterpart, as shown in Figure 1.

The origin of the wake-up effect of the RTA MFM capacitors is generally attributed to the built-in field bias caused by the redistribution of defects, particularly the oxygen vacancies [13,16]. Owing to the heating effect during the RTA process, the nitrogen and titanium ions of the TiN electrodes diffuse into the HZO film, and the TiN electrodes get partially oxidized, thus generating the oxygen vacancies at the interfaces of the HZO ferroelectric layer and TiN electrodes. The HZO film is polycrystalline with a granular structure having numerous *V*_O_ defects at the grain boundaries. The charge trapping in the interfacial and bulk defects may lead to a local electric field and pinning of the partial polarization, which will be released during cycling [16]. It can be observed from the former experimental results that the samples annealed by the MWA treatment exhibit a suppressed wake-up effect since the MWA-induced electric fields limit the formation of defects and redistribution during the annealing.

**Figure 5:**
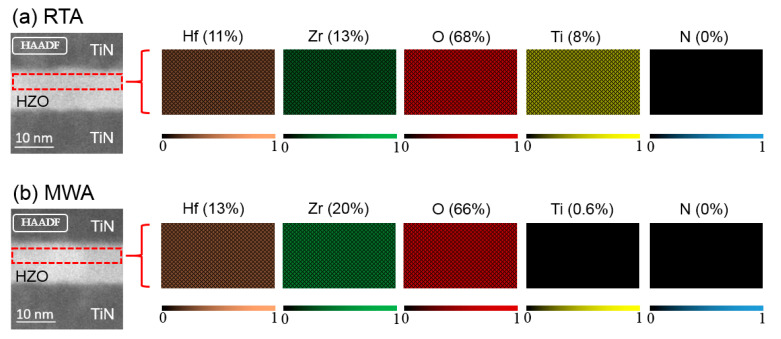
False-color EDX maps and high-angle annular dark-field (HAADF) images represent the average composition of the HZO films with (**a**) MWA and (**b**) RTA treatment.

**Figure 6:**
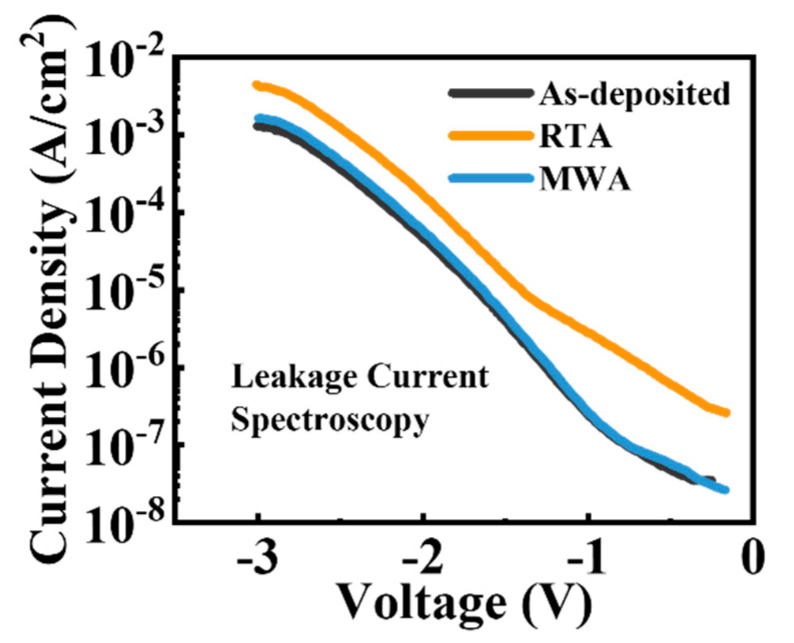
Leakage current density characteristics under different voltages of the as-deposited, RTA, and MWA samples.

Figure 5 displays the EDX spectroscopy elemental maps of the RTA and MWA capacitors (see detailed EDX examinations in Figure S2 in the Supplementary Information). Ti concentration is 8% of the RTA sample in the selected region, whereas that of the MWA capacitor is merely 0.6%, indicating the significantly mitigated interfacial diffusion in the MWA capacitor. The leakage current can reflect the quantities of defects. Meanwhile, high leakage currents may affect the time-dependent dielectric breakdown (TDDB) stability [33], which is detrimental to logic device applications and hinder further scaling of ferroelectric technologies. Figure 6 shows the leakage current characteristics of the HZO MFM capacitors subjected to the different annealing conditions at a negative bias from 0 V to −3 V. The capacitor with the MWA treatment presents a comparable leakage current density as the as-deposited counterpart. Notably, the leakage current densities of the MWA device are smaller than those of the RTA device, indicating a smaller amount of bulk defects *V*_O_ in the HZO film of the MWA device. 

MWA is more efficient than RTA for suppressing defects due to the unique electromagnetic properties [22,23,24] of MWA. During the RTA process, the thermal energy is transferred from the outer surface to the inside of the device. Therefore, it will generate a temperature gradient that results in inefficient energy consumption, potential interfacial diffusion, and additional defects [16]. Nevertheless, in the MWA process, the electromagnetic field selectively exerts a force on the VO’s dipoles, transfers energy directly to the local defects, and anneals without inducing strong diffusion in other regions. Therefore, the MWA devices exhibit better ferroelectric properties with a larger Pr, lower wake-up effect, and smaller leakage current.

## 4. Conclusions

The effects of the different annealing treatments of the ferroelectric HZO MFM capacitors were investigated and analyzed by comparing MWA to the widely applied RTA. The electrical characteristic measurements, including P-V, I-V, electric field cycling, and characterization techniques of HRTEM, GIXRD, and EDX, were conducted to investigate the physical properties comprehensively. First, the orthorhombic phase is observed in both the MWA and RTA HZO films. By being subjected to the MWA treatment, the HZO MFM capacitors exhibit better ferroelectric properties than the RTA counterparts, and the 2Pr value reaches 63 µC/cm^2^. Besides, the devices subjected to MWA exhibit a lower wake-up effect, and the parameter *R* (characterizing the change of 2Pr) is three times lower than that of the RTA devices. Furthermore, the devices annealed by MWA treatment have smaller leakage current density and better interface state density due to the inhibited interdiffusion of the TiN and HZO, characterized by the EDX spectroscopy. The suggested MWA method is foreseen as a potential alternative annealing solution to enhance the ferroelectric properties of the HZO thin films of low-cost, high efficiency, and full CMOS compatibility.

## Data Availability

Not applicable.

## Supplementary Materials

The following supporting information can be downloaded at: https://www.mdpi.com/article/10.3390/nano12173001/s1. Detailed discussions about the factors facilitating the formation of the ferroelectric phases, the HRTEM characterization, and the EDX elemental analysis are described in the Supplementary Information. Figure S1. The EDX spectrum of the RTA sample; Figure S2. The EDX spectrum of the MWA sample. Refs. [34,35] are cited in Supplementary Materials.
