# Supplementary material for: Improved Ferroelectric Properties in Hf0.5Zr0.5O2 Thin Films by Microwave Annealing
Source: Nanomaterials (Basel). 2022 Aug 30;12(17):3001. doi: 10.3390/nano12173001 (PMC9457976; doi:10.3390/nano12173001)
Supplement: Supplementary file 1 [file nanomaterials-12-03001-s001.zip › nanomaterials-1859721-supplementary.pdf]

nanomaterials

Supplementary Materials

Improved Ferroelectric Properties in $Hf_{0.5}Zr_{0.5}O_2$ Thin Films by Microwave Annealing

S1. Factors facilitating the formation of the ferroelectric phase

For hafnium-based thin films, the outstanding ferroelectric (FE) properties with large remanent polarization (Pr) values are mainly attributed to the polar orthorhombic phase ($Pca2_1$, o-phase). However, the nonpolar phases usually coexist with the polar phase, such as the monoclinic phase (P21/c, m-phase) and tetragonal phase (P42/nmc, t-phase). A higher volume fraction of polar o-phase and lower nonpolar fraction will result in better ferroelectric properties. The polar o-phase is stabilized and transformed from the t- and m-phases of metastability, which relies on several extrinsic factors, including the capping electrodes, dopants, and grain size effects [1–4]. The capping of top and bottom electrodes can provide mechanical stress, which is indispensable for the crystallization of ferroelectric phases. Moreover, doping is essential in hafnium-based ferroelectric films to reduce the stability of the nonpolar m-phase. Among the reported dopants (Si, Zr, Y, Al, Gd, Sr, La), Zr is the most promising due to Hf's similar physical and chemical properties and the relatively low crystallization temperature of ZrO_2 [3]. In addition, the grain size or surface energy effect is another influential factor. The FE o-phase can be stabilized by optimizing the grain size and the surface energy by adjusting the HZO film thickness. It is well documented in the previous studies that the Zr-concentration of about 50% and film thickness of less than 20 nm are the optimum conditions to achieve better ferroelectricity in hafnia films [4]. With these above considerations, the TiN top and bottom electrodes, 50% Zr doping concentration, and 10 nm thickness HZO film were identically adopted in both microwave annealing (MWA) and rapid thermal annealing (RTA) samples

S2. Detailed EDX elemental analysis

The EDX spectroscopy was performed to investigate the diffusion of elements from the metal cap layer into the ferroelectric HZO film using an FEI-Titan instrument operating at an acceleration voltage. The elemental maps were obtained in the ChemSTEM mode using a Bruker EDS windowless silicon drift detector (SDD) concurrently with high-angle annular dark-field (HAADF) images. The point spectrum was collected in the selected regions of the bottom TiN electrode, the HZO film, and the top TiN electrode. For the HZO films in both samples, the collection regions were located near the top TiN/HZO interfaces to examine the diffuseness of the interface. The concentrations of each element are shown in Figure S1 and S2, respectively. For RTA samples shown in Figure S1, it offers an ~8% Ti concentration in the selected region of HZO film, indicating a clear trace of severe interdiffusion and the formation of interfacial defects. In contrast, in the MWA sample depicted in Figure S2, the Ti concentration is only 0.6% (Figure S2), illustrating less interdiffusion and fewer interfacial defects than the RTA counterpart.

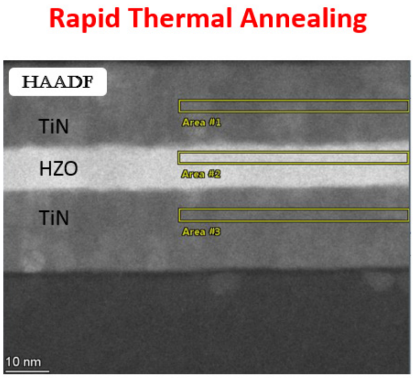

Sample	Layer	Element	Concentration(%)
RTA	TiN	Ti	53.66
		N	46.34
	**HZO**	**Hf**	**10.98**
		**Zr**	**12.96**
		**O**	**68.2**
		**Ti**	**7.86**
		**N**	**0**
	TiN	Ti	42.71
		N	57.29

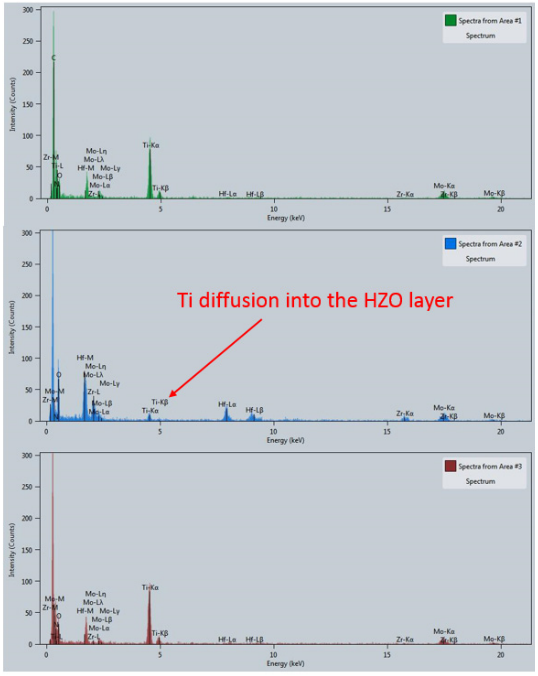

Figure S1. The EDX spectrum of the RTA sample.

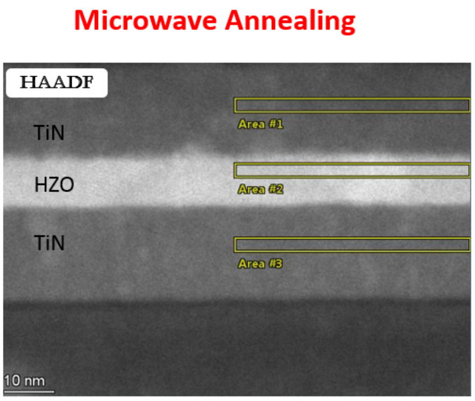

Sample	Layer	Element	Concentration(%)
MWA	TiN	Ti	40.49
		N	59.51
	**HZO**	**Hf**	**12.8**
		**Zr**	**20.29**
		**O**	**66.32**
		**Ti**	**0.59**
		**N**	**0**
	TiN	Ti	56.71
		N	43.29

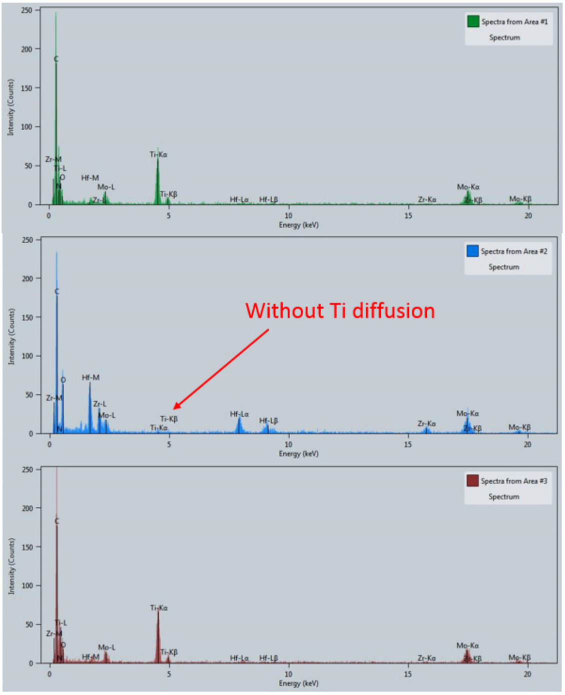

Figure S2. The EDX spectrum of the MWA sample.

References

[1] Park, M.H.; Lee, Y.H.; Kim, H.J.; Schenk, T.; Lee, W.; Kim, K.D.; Fengler, F.P.G.; Mikolajick, T.; Schroeder, U.; Hwang, C.S. Surface and Grain Boundary Energy as the Key Enabler of Ferroelectricity in Nanoscale Hafnia-Zirconia: A Comparison of Model and Experiment. *Nanoscale* **2017**, *9*, 9973–9986. https://doi.org/10.1039/C7NR02121F.
[2] Pešić, M.; Fengler, F.P.G.; Larcher, L.; Padovani, A.; Schenk, T.; Grimley, E.D.; Sang, X.; LeBeau, J.M.; Slesazeck, S.; Schroed-er, U.; et al. Physical Mechanisms behind the Field-Cycling Behavior of HfO2 -Based Ferroelectric Capacitors. *Adv. Funct. Ma-ter*. **2016**, *26*, 4601–4612. https://doi.org/10.1002/adfm.201600590.

[3] Takahisa, S.; Kiliha, K.; Tatsuhiko, Y.; Takao, S.; Takahiro, O.; Osami, S.; Hiroshi, U.; Yasuhiko, I.; Takanori, K.; Toyohiko, J.K. and Hiroshi, F. Impact of mechanical stress on ferroelectricity in $(Hf_{0.5}Zr_{0.5}) O_2$ thin films. *Applied Physics Letters*, **2016**, *108*, 262904.
[4] Kim, S.J.; Mohan, J.; Summerfelt, S.R.; Kim, J. Ferroelectric $Hf_{0.5}Zr_{0.5}O_2$ thin films: a review of recent advances. *JOM*, **2019,** *71*, 246–255.
